# Powdered tart cherry supplementation demonstrates benefit on markers of catabolism and muscle soreness following an acute bout of intense lower body resistance exercise

**DOI:** 10.1186/1550-2783-11-S1-P31

**Published:** 2014-12-01

**Authors:** K Levers, R Dalton, E Galvan, C Goodenough, A O’Connor, S Simbo, N Barringer, J Carter, C Seesselberg, YP Jung, A Coletta, S Mertens-Talcott, C Rasmussen, M Greenwood, R Kreider

**Affiliations:** 1 Texas A&M University, College Station, Texas, USA

## Background

Consumption of tart cherry juice has been reported to effectively reduce inflammation, muscle damage, and muscle soreness following bouts of exercise. The purpose of this study was to determine if consumption of a powdered form of tart cherries derived from tart cherry skins prior to and following intense resistance exercise promotes similar positive results as seen with tart cherry juice consumption.

## Methods

23 resistance trained men (20.9±2.6 yr, 14.2±5.4% body fat, 63.9±8.6 kg FFM) volunteered to participate in this study and were matched based on relative maximal back squat strength, age, body weight, and fat free mass. Subjects were randomly assigned to ingest, in a double blind manner, capsules containing a placebo (P, n=12) or powdered tart cherries (CherryPURE® Freeze Dried Tart Cherry Powder [TC, n=11]). Participants ingested the supplements one time daily (480 mg/d) for 10-d including day of exercise up to 48-hr post-exercise. Participants performed 10 sets of 10 repetitions at 70% of 1RM barbell back squat with 3 minutes recovery between sets, maintaining equivalent average work values between groups throughout the protocol (p=0.24). Participants rated perceptions to a standardized application of pressure via an algometer on the dominant thigh at 3 designated locations using a 10-point visual analogue scale to assess muscle soreness/tenderness over the course of the testing protocol. Fasting blood samples and VAS ratings of muscle soreness were taken pre-squat workout, 60-minutes following the squat workout as well as after 24 and 48 hours of recovery and analyzed by MANOVA with repeated measures. Consent to publish the results was obtained from all participants.

## Results

Pain ratings from all 3 quadriceps locations (p<0.001); AST, ALT, CK, BUN/Cr ratio, UA (p<0.001); cortisol, testosterone, and cort/test ratio (p<0.001) all demonstrated significant changes in both groups over time, but the overall Wilks’ Lambda MANOVA analysis did not reveal a significant group x time effect for any pain ratings (p=0.199); AST, ALT, CK, BUN/Cr ratio, UA (p=0.605); cortisol, testosterone, CORT/TEST ratio (p=0.35). MANOVA univariate analysis revealed significant time effects for all locations of pain ratings (p<0.001); AST, UA (p<0.001); cortisol, testosterone (p<0.001); CK (p=0.003); and CORT/TEST ratio (p=0.022) in both groups in addition to trends for both groups over time for ALT (p=0.094) and BUN/Cr ratio (p=0.059). A significant group x time quadratic effect was shown for v. lateralis [¼] pain perception (p=0.024) with a trend toward a significant cubic interaction for the v. lateralis [½] pain perception (p=0.10). No significant group x time interaction was evident in pain perception from the v. medialis [¼] (p=0.24), but a delta value trend was shown based on v. medialis [¼] pain perception differences in group assignment (p=0.10). A significant group x time linear effect was shown for UA (p=0.014) along with a trend toward a significant linear interaction (p=0.067) and a significant delta value based on group assignment for ALT (p=0.005). A trend toward a significant group x time linear effect was shown for cort/test ratio (p=0.10). No significant group x time interactions were evident for cortisol (p=0.45) and testosterone (p=0.17).

## Conclusion

Results of this study indicate that acute supplementation with powdered tart cherries over the 7 days leading up to, during, and 2 days after intense resistance exercise helps to minimize post-training perceptions of pain in the most biomechanically loaded regions of the quadriceps muscle group associated with the back squat compared to a placebo. Additionally, powdered tart cherry supplementation helped attenuate the hepatic response of ALT and catabolic/antioxidant response of UA following a bout of intense resistance exercise. Overall, these findings suggest that supplementation with a powdered tart cherry product surrounding an intense resistance event reduces pain perception in addition to hepatic and catabolic stress in the post-exercise period. Further research is necessary to determine long-term supplementation effects with resistance training.

